# The Abandoned Radical Hysterectomy for Cervical Cancer: Clinical Predictors and Outcomes

**DOI:** 10.1155/2010/743794

**Published:** 2010-04-28

**Authors:** Heidi J. Gray, Erin Seifert, Victor G. Sal y Rosas, Katrina F. Nicandri, Wui-Jin Koh, Barbara A. Goff

**Affiliations:** ^1^Department of Obstetrics and Gynecology, University of Washington School of Medicine, Seattle, WA 98195, USA; ^2^Department of Biostatistics, University of Washington, Seattle, WA 98195, USA; ^3^Department of Obstetrics and Gynecology, University of Rochester School of Medicine, Rochester, NY 14642, USA; ^4^Department of Radiation Oncology, University of Washington, Seattle, WA 98195, USA

## Abstract

*Objective*. Cervical cancer patients who had an abandoned radical hysterectomy were evaluated for preoperative clinical predictors, complication rates, and outcomes. *Study Design*. IRB approval was obtained for this retrospective analysis and chart review was performed. *Results*. From 268 women with early-stage (IA2 to IIA) cervical cancer, 19 (7%) had an abandoned hysterectomy for finding grossly positive lymph nodes (84%) or pelvic spread of tumor (16%). No clinical characteristics clearly identified women preoperatively at risk of having an abandoned hysterectomy. In the abandoned group, 26% suffered major morbidities, compared to 34% in the completed group (OR 0.69, [CI 0.16–2.57], *P* = .789). Thirty-seven percent recurred in the abandoned group, compared to 18% in the completed group (*P* = .168). Overall survival in the abandoned group was 73% versus 80% in the completed group (*P* = .772). *Conclusion*. The practice of abandoning a planned radical hysterectomy for unexpected metastatic disease may not worsen the outcome.

## 1. Introduction

Cervical cancer affects a large number of women with an estimated 450,000 new cases per year globally; approximately 10,000 are diagnosed annually in the U.S. [1]. The vast majority of women will be diagnosed at early clinical stage when cure rates are high. Early-stage (IA2 to IIA) carcinoma of the cervix can be treated by radical hysterectomy (RH) or primary radiotherapy (RT) with similar outcomes (five-year overall survival 87%–92%) [2]. The choice of therapeutic modality is based on patient comorbidities, and patient or physician preference. Benefits to surgical treatment include simultaneous lymphadenectomy for surgical staging [3] with possible therapeutic benefit [4], preservation of ovarian function in premenopausal women, and improved coital function, as compared to RT [5–7].

However, a subset of women treated with primary surgery will require adjuvant postoperative chemoradiation due to findings which confer a high risk of recurrence. These include cancer spread to lymph nodes, invasion into the parametria, and positive surgical margins [8]. In order to avoid the combined morbidity of both methods of treatment, many surgeons will abandon the radical hysterectomy intraoperatively (termed abandoned or aborted radical hysterectomy) if there are findings of disseminated disease, such as positive lymph nodes. This occurs in approximately 8%–10% of radical hysterectomies for early-stage cancer [9, 10], and primary treatment with chemoradiation follows. This is an area of controversy in gynecologic oncology, and there is no consensus regarding the most appropriate management. Further, data on preoperative identification of women who may be at risk of an abandoned hysterectomy is lacking. 

The finding of extra-cervical spread at the time of exploration for radical surgery presents a challenging decision: proceed with planned hysterectomy knowing that the patient will require further postoperative therapy or abandon the procedure for primary chemoradiation. Therefore, we embarked on a review of cervical cancer patients at our institution that had an abandoned radical hysterectomy for preoperative clinical characteristics, morbidities and survival. 

## 2. Materials and Methods

IRB approval was obtained to review charts of patients who underwent surgical exploration for early-stage cervical cancer (Stage IB1-IIA) for the intent of radical hysterectomy over a 10-year period at the University of Washington Medical Center. We identified patients whose planned hysterectomy was abandoned due to intraoperative findings of metastatic spread and abstracted data from medical records on demographics, laboratory values, preoperative radiologic imaging, operative reports, pathology specimens, complications, recurrence, and overall survival. In addition, we identified a group of early-stage patients who underwent a radical hysterectomy and were found to have the high risk feature of positive lymph nodes postoperatively to compare morbidities and outcomes of therapy (completed group). Statistical analysis was performed by Fisher's exact method between two variables and Log rank test to compare survival probabilities. 

## 3. Results

Between 1993 and 2003, 268 women with early-stage (IA2 to IIA) cervical cancer presented for primary surgical management with radical hysterectomy at our institution. On review of operative reports, nineteen patients (7%) had intraoperative abandonment of their planned radical hysterectomy. The median age was 42 years old (range 29–85) and approximately half were smokers (*n* = 9, 47%) with median pack year history of 18. Most were stage IB1 (63%) and squamous histology (79%) (Table 1). 

Looking at other preoperative characteristics, the most common presenting symptom was vaginal bleeding (*n* = 12, 68%), whereas only three (16%) of patients were diagnosed following referral for an abnormal Pap smear. The median number of years since prior Pap smear was four (range 1–60). Median preoperative hematocrit and hemoglobin were 34.5 and 12.0, respectively (range 29–41, 9.6–14.1). Preoperative imaging by CT scan was obtained in 12/19 (63%). Of these, 50% (*n* = 6) had findings suspicious for pelvic lymphadenopathy (Table 1). No patients had preoperative PET imaging during this study period. 

The reasons for abandonment were positive pelvic lymph nodes (84%), positive paraaortic lymph nodes (16%) and/or peritoneal spread (16%) by intraoperative frozen section. Of the 16 patients who had abandonment for positive pelvic lymph nodes, 25% were found to have positive para-aortic or common iliac lymph nodes on final pathology. In total, 7 of 19 (37%) had positive para-aortic or common iliac lymph nodes. The mean number of lymph nodes removed was 23 (17 pelvic and 6 para-aortic, range 0–82) and the mean number of positive lymph nodes was 2.7 (range 0–9). 

Most women received definitive postoperative chemoradiation therapy (12/19, 63%) or radiation alone (4/19, 32%). One patient with adenocarcinoma spread to scalene nodes underwent palliative chemotherapy and died of disease within 12 months. Five patients were treated with concurrent 5-FU and cisplatin, one with gemcitbine and cisplatin, and six with cisplatin alone. The median radiation dose was 45 Gy (range 30.6–50.4) external beam whole pelvic and 85 Gy (range 71–93) to point A with either LDR or HDR. Following radiation therapy, only three patients (16%) underwent an adjuvant hysterectomy, and one (33%) had residual disease. 

We compared clinical characteristics, morbidity, and mortality to a group of early-stage patients (*n* = 44) during the same time period that had a completed radical hysterectomy, but were found postoperatively to have positive pelvic lymph nodes (completed group). All received external beam radiation or chemoradiation following radical surgery, without brachytherapy. They were similar in demographic makeup such as age, stage, and histology (Table 1). However, they were more than twice as likely to present with an abnormal Pap smear (32%), and time from prior Pap smear was median 2.5 years. Other preoperative characteristics such as weight and hemoglobin were not appreciably different between the two groups (Table 1). 

Next, we evaluated major complications, both operative-and radiation-related. There were five major complications in the abandoned group (26%): two operative-related (intraoperative stroke and bowel perforation postoperatively) and three radiation-related (severe lymphedema, severe radiation proctitis, requiring colostomy, and rectovaginal fistula). In the completed group, there were fifteen major complications (34%), five operative and 10 radiation-related (Table 2). The difference in morbidity was not significant. 

**Figure 1:**
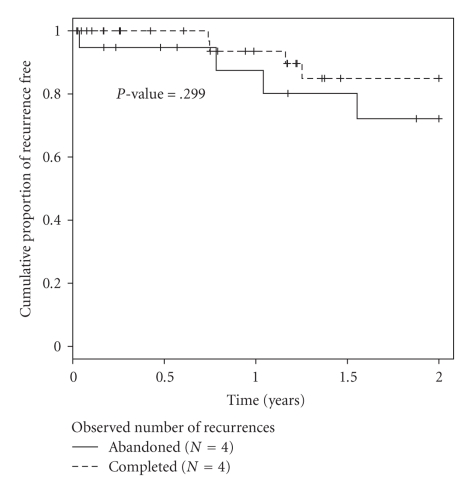
Cumulative probability of recurrence free at two years of followup.

**Figure 2:**
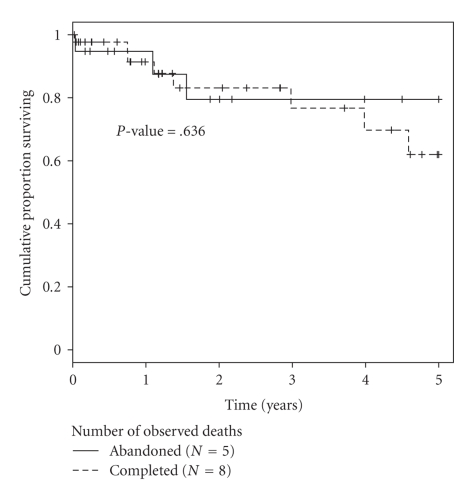
Overall survival at five years for abandoned hysterectomy and completed hysterectomy groups.

In the abandoned group, there were seven patients (37%) who experienced recurrence. Three (43%) had positive para-aortic or common iliac lymph nodes, in addition to positive pelvic lymph nodes. Recurrence site was pelvic in four patients and distant in three. Of those with recurrence, only two (29%) were able to be salvaged with exenteration and five (71%) have died. Overall survival rate is 73% after two years follow-up. In the completed group, eight patients had recurrence (18%). Recurrence site was pelvic in four and distant in four. Of this group, none were are able to salvaged and one died of other causes, for an overall survival of 80%. By Kaplan-Maier estimates, the progression-free and overall survival difference between the two groups is not significant at the twoyear interval (Figures 1 and 2).

## 4. Comment

In our series of 268 women with early-stage cervical cancer, 19 (7%) had an abandoned hysterectomy when unexpected spread of disease was found outside the cervix at time of surgical exploration. We found patients with abandoned hysterectomy presented more often with vaginal bleeding (68%), as opposed to an abnormal Pap (16%), which suggests that may the disease be further along in the pathogenesis. Additionally, published reports have documented length of time from prior Pap smear as an association with more advanced disease and poorer outcome [11]. In our group of patients who had an abandoned hysterectomy, the average time from prior Pap smear was four years. Therefore, increasing time from prior Pap smear may also be an indicator of finding metastatic spread at time of surgical exploration. 

The association of anemia and poor prognosis in patients with cervical cancer has been well established in the literature [12–14]. We therefore hypothesized that women at risk of an abandoned hysterectomy may be anemic on presentation as a marker of more advanced disease. However, median preoperative hemoglobin and hematocrit were not markedly low in our group. 

The use of preoperative imaging with CT or PET-CT is increasingly becoming the standard of care in the US to help identify metastatic disease [15, 16]. Although our study cohort spanned a time prior to more widespread preoperative imaging (only 63% of patients received), we identified suspicious lymphadenopathy in 50% of the patients who underwent a CT scan. Suspicious lymph nodes on CT alone are typically reported by size criteria as greater than 10 mm. Therefore, lymph nodes that have normal size may still harbor “microscopic” spread and will be missed on CT. PET-CT may be able to overcome some of these limitations and currently is approved for preoperative workup of metastatic disease in the US. Overall PET-CT has been shown to have a sensitivity and specificity of 75% and 96% for detecting nodal metastasis [17]. However, some investigators have found lower sensitivity in women with early-stage disease [18], but we still recommend patients with newly diagnosed invasive cervical cancer to undergo a PET-CT to aid in treatment planning if feasible. 

Despite best efforts to identify preoperatively patients who have metastatic disease, there will still be patients who on surgical exploration have metastatic disease. Controversy exists as to the best management; whether to proceed with radical hysterectomy or abandon for definitive chemoradiation. When there is disseminated disease to the peritoneum or abdomen, the prognosis is poor enough that there is little doubt that radical surgery will contribute significantly to improving outcome. Additionally, the finding of grossly positive para-aortic lymph nodes confers worse prognosis with five year overall survival of rates of 30%–40%. Performing radical surgery in this setting is unlikely to improve and may delay definitive treatment for recovery time. However, it is recommended to attempt surgical resection of grossly enlarged lymph nodes to improve radiation response and possibly improve survival [4, 19]. 

The finding of positive pelvic lymph nodes when exploring for radical surgery presents a unique dilemma. There are no prospective clinical trials randomizing patients into abandoned versus completed radical hysterectomy when unexpected metastatic disease to pelvic lymph nodes is found. Proponents for abandoning argue that patients suffer less radiation toxicity to small bowel, rectum, and bladder when the uterus is left in place, and have shorter interval to recovery and definitive treatment. Those in favor of completing the hysterectomy argue that removal of the primary tumor may reduce the recurrence risk with better pelvic control and allow for improved survival. 

The first report that described the abandoned (or aborted) radical hysterectomy in cervical cancer was from 1990, in which 15 women with aborted radical hysterectomy were matched with 15 women who had completed hysterectomy, but were found to have positive nodes on pathologic examination after surgery. They excluded all patients with positive para-aortic lymph nodes. Still, survival rate was 45% in the abandoned group versus 30% in the completed group, with only one case of radiationrelated morbidity (7%), which is low compared to most reports [9]. Hopkins and Morley reported in 1991 on 14 patients that had an abandoned hysterectomy, mostly for positive lymph nodes, (57%) and compared to a group of 26 patients found to have involved lymph nodes postoperatively. The overall survival was 50% in the abandoned group compared to 70% in the completed. Morbidities were higher (20%) for the completed compared to the aborted group(16%) [20]. 

In total, there have been seven retrospective series of patients with abandoned hysterectomies reported in the literature. Table 3 summarizes major findings between the studies [9, 10, 20–24]. In general, the reported overall survival ranges from 31%–83% at five years. The earlier studies have a lower survival rate possibly due to lack of chemotherapy paired with radiation. The reported morbidities vary greatly (range 7 to 48%) and are fraught with reporting error, as these all are retrospective and patients are commonly lost to follow-up. 

In our study, we compared patients that had an abandoned hysterectomy to a group of women that had undergone radical hysterectomy, but had positive lymph nodes postoperatively for morbidities, recurrence rates and survival. There are clear limitations to using this control group, as women found to have only microscopically positive lymph nodes would inherently be expected to have higher survival rates than women with gross or suspicious lymph nodes. In our abandoned group, major toxicities related to treatment were common (26%), but lower in the completed group (34%), although this was not statistically significant. The increase appeared to be mostly due to increased severe radiation-related toxicity (16% versus 23%). Only two prior studies have reported on morbidity in the completed (surgery + radiation) group, which ranged from 22%–29% [20, 24]. One limitation of all of these retrospective studies, including ours, is that the reporting of morbidity is not standardized and can be underreported. 

Progression free survival in our abandoned group was lower than what would be expected for early-stage disease (63%), although several patients were able to be salvaged by exenteration for an overall survival of 73%. Our findings were similar to previously reported studies (Table 3). A major limitation of this study is that it was underpowered to detect a difference in survival between the abandoned group and completed group. 

In conclusion, individuals with clinically early-stage cervical cancer who present with bleeding or long interval from prior pap smear should be considered at risk for more advanced disease. Preoperative imaging with CT or PET-CT may help to evaluate for metastatic spread. If faced with unexpected metastatic spread to pelvic lymph nodes at time of surgical exploration, many surgeons will choose to abandon the radical hysterectomy for primary chemoradiation therapy. However, completion of the hysterectomy in this setting may not worsen morbidity or effect overall survival. As it is unlikely there will ever be a prospective randomized trial to fully answer this question, our study suggests that either option is viable and should be discussed with the patient during the preoperative counseling. 

## Figures and Tables

**Table 1:** Patient characteristics.

	Abandoned (*n* = 19)	Completed (*n* = 44)	*P*
*Age (Years)* ^#^	42 [41–54, 29–85]	44 [37–54, 21–85]	.4207
*Weight (kg)* ^#^	83 [65–85, 42–85]	71 [60–86, 41–121]	.8727
*Smoker (Yes)*	9 (47%)	27 (61%)	.4515
Pack per year^#^	18 [10–30, 2–52]	20 [10–30, 2–75]	.682

*Stage*			.4999
IB	0 (0%)	3 (7%)	
IB1	12 (63%)	29 (66%)	
IB2	5 (26%)	10 (23%)	
IIA	1 (5%)	2 (5%)	
IIB	1 (5%)	0 (0%)	

*Histology*			.6747
Squamous	14 (74%)	31 (70%)	
Adenocarcinoma	4 (21%)	12 (27%)	
Adenosquamous	1 (5%)	1 (2%)	

*Presenting symptoms*			.4407
Abnormal pap	3 (16%)	14 (32%)	
Vaginal bleeding	13 (68%)	23 (52%)	
Other	3 (16%)	3 (16%)	

*Preoperative labs*			
Hemoglobin^#^	12 [11.8–12.7, 9.6–14]	12.7 [12.1–13.4, 7.8–14.2]	.3385
Hemotocrit^#^	34.5 [32–37, 29–41]	38 [35.8–39.3, 22–44]	.2456

*Prior pap (Years)*	4 [2–9, 1–60]	2.5 [1–6,1–25]	.2691
*Preoperative CT scan* ^⋆^			
No	7 (37%)	NR	
Yes	12 (63%)	NR	
Suspicious	6 (50%)	NR	

^#^Median [IQR  =  interquartile Interval, Range  =  Minimum-Maximum].

^⋆^NR  =  not recorded.

**Table 2:** Surgical-and radiation-related complications.

Complication	Abandoned (*N* = 19)	Completed (*N* = 44)	OR, 95% CI and *P*-value
*Surgical*			
Stroke	1	—	
Bowel perforation/obstruction	1	1	
Hemorrhage (>1500 cc)	—	1	
Ureteral injury	—	1	
Iliac vein laceration	—	1	
Neurogenic/denervated bladder	—	1	

Total surgical complications	2/19 = 10%	5/44 = 11%	(0.92, [0.08–6.633] and 1)

*Radiation-related*			
Lymphedema	1	3	
Radiation enteritis/proctitis	1	2	
Bowel obstruction	—	1	
Severe diarrhea requiring TPN	—	1	
Rectovaginal fistula	1	—	
Radiation cystitis	—	2	
Vaginal stenosis	—	1	

Total radiation complications	3/19 = 16%	10/44 = 23%	(0.64, [0.10–2.98] and 0.738)

Total	5/19 = 26%	15/44 = 34%	(0.69, [0.16–2.57] and 0.789)

Note: All complications were grade 3 or higher. Some patients had more than one complication.

**Table 3:** Summary of published reports of abandoned hysterectomy.

Author	Stage	N	Morbidity^+^	Recurrence	Survival
Potter et al. [9]	IB-IIA	15	7%	53%	45%–50%
Hopkins and Morley [20]	IB-IIA	14	16%	NR	50%
Bremer et al. [21]	IB-IIA	26	45%	39%	61%
Whitney and Stehman [22]	IB	68	19%	65%	31%
Leath et al. [23]	IB-IIA	23	34%	26%	83%
Suprasert et al. [24]	IB-IIA	23	48%	26%	59%
Richard et al. [10]	IB	55	NR	NR	71%
Gray 2010	IB-IIA	19	26%	37%	73%

Total Abandoned		243	7%–48%	26%–65%	31%–83%

^+^Morbidities are combination of surgical-and-radiation related.
